# New Generation of Osteoinductive and Antimicrobial Polycaprolactone-Based Scaffolds in Bone Tissue Engineering: A Review

**DOI:** 10.3390/polym16121668

**Published:** 2024-06-12

**Authors:** Bartolomeo Coppola, Francesca Menotti, Fabio Longo, Giuliana Banche, Narcisa Mandras, Paola Palmero, Valeria Allizond

**Affiliations:** 1 Department of Applied Science and Technology, Politecnico di Torino, 10129 Turin, Italy; bartolomeo.coppola@polito.it (B.C.); paola.palmero@polito.it (P.P.); 2 Department of Public Health and Pediatrics, University of Torino, 10126 Turin, Italy; francesca.menotti@unito.it (F.M.); narcisa.mandras@unito.it (N.M.); valeria.allizond@unito.it (V.A.)

**Keywords:** scaffolds, polycaprolactone, calcium phosphates, antimicrobial agents, metal ions, essential oils, bacterial adhesion, biofilm formation, eukaryotic cell proliferation and integration

## Abstract

With respect to other fields, bone tissue engineering has significantly expanded in recent years, leading not only to relevant advances in biomedical applications but also to innovative perspectives. Polycaprolactone (PCL), produced in the beginning of the 1930s, is a biocompatible and biodegradable polymer. Due to its mechanical and physicochemical features, as well as being easily shapeable, PCL-based constructs can be produced with different shapes and degradation kinetics. Moreover, due to various development processes, PCL can be made as 3D scaffolds or fibres for bone tissue regeneration applications. This outstanding biopolymer is versatile because it can be modified by adding agents with antimicrobial properties, not only antibiotics/antifungals, but also metal ions or natural compounds. In addition, to ameliorate its osteoproliferative features, it can be blended with calcium phosphates. This review is an overview of the current state of our recent investigation into PCL modifications designed to impair microbial adhesive capability and, in parallel, to allow eukaryotic cell viability and integration, in comparison with previous reviews and excellent research papers. Our recent results demonstrated that the developed 3D constructs had a high interconnected porosity, and the addition of biphasic calcium phosphate improved human cell attachment and proliferation. The incorporation of alternative antimicrobials—for instance, silver and essential oils—at tuneable concentrations counteracted microbial growth and biofilm formation, without affecting eukaryotic cells’ viability. Notably, this challenging research area needs the multidisciplinary work of material scientists, biologists, and orthopaedic surgeons to determine the most suitable modifications on biomaterials to design favourable 3D scaffolds based on PCL for the targeted healing of damaged bone tissue.

## 1. Introduction

In the human body, bone is a connective tissue characterised by a high specialisation, vascularisation, and mineralised extracellular matrix. It serves vital functions, specifically providing strength, rigidity, defence, and sustenance to the tissues, while also serving as a reserve of calcium and phosphates [1]. In natural conditions, well-balanced bone tissue remodelling is crucial for the skeletal system’s homeostasis. Three types of eukaryotic cells are involved in its resorption, preservation, and neoformation: osteoblasts, osteocytes, and osteoclasts [2]. When a trauma, a tumour, osteoporosis, or other external factors cause a bone defect, physiological healing occurs only if a small tract is involved; conversely, extended bone tract damage is not restored by the body, and a fibrous connective mass is deposed [3,4]. Therefore, it is really necessary that suitable restoration is promoted by a bone graft created using biological or synthetic materials [5,6]. The gold standard for restoring lost bone is an autologous graft; however, due to the risk of morbidity in the donor and the progress made in tissue engineering research, bone tissue implants have become a suitable solution for many patients [1,7].

The key aspect of bone tissue engineering is the production of a temporary three-dimensional (3D) structure able to allow the reconstruction of the damaged bone tissues, using a scaffold based on various polymers that can be modified to impart and to improve different properties [4]. A pivotal aspect of biomaterial design is to facilitate the process of tissue regeneration in the site of bone loss and, thereafter, being “resorbed and substituted” by new bone tissue deposition [1]. Additionally, the 3D scaffold should have high and interconnected porosity. Polymers are utilised as bone implants due to various characteristics, mainly biocompatibility, modelling elasticity, low weight, and ductility, but they present low stiffness [5].

Polymers can be categorised as natural or synthetic; the former encompass chitosan, col, collagen, and alginate, whereas the latter include polycaprolactone (PCL), poly (lactic acid) (PLA), and poly (lactic-co-glycolic) acid (PLGA). Chitosan displays a non-toxic nature and biodegradability, although its mechanical strength could be improved by the addition of hydroxyapatite (HA) [1]. Col is biocompatible and allows for bone cell adhesion and proliferation, and it is not antigenic, even if it has a fast rate of degradation and low mechanical strength that could be enhanced with the addition of HA. Due to the complexity in modifying natural polymers, the utilisation of synthetic ones has become increasingly necessary.

**Figure 1:**
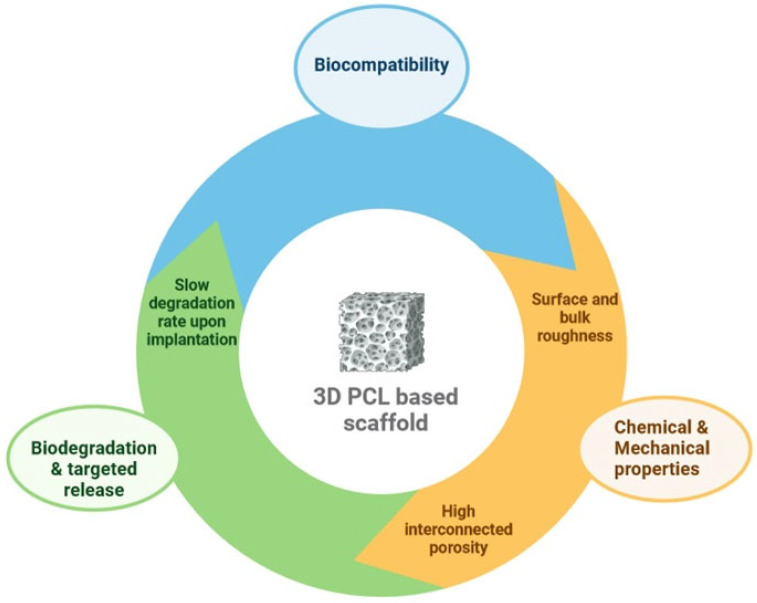
Key features of pure PCL for bone tissue engineering.

In this context, PCL, a synthetic polymer, has outstanding properties in tissue engineering, including compatibility with osteoblasts, bioresorbability, a semicrystalline nature, and a non-cytotoxic effect towards eukaryotic cells, making it an appropriate biomaterial to guide bone regeneration [8,9,10]. Additionally, PCL is a cost-effective raw material when compared with other biodegradable polymers [11]. Figure 1 shows the properties of pure PCL that make it a perfect candidate in bone tissue engineering.

**Figure 2:**
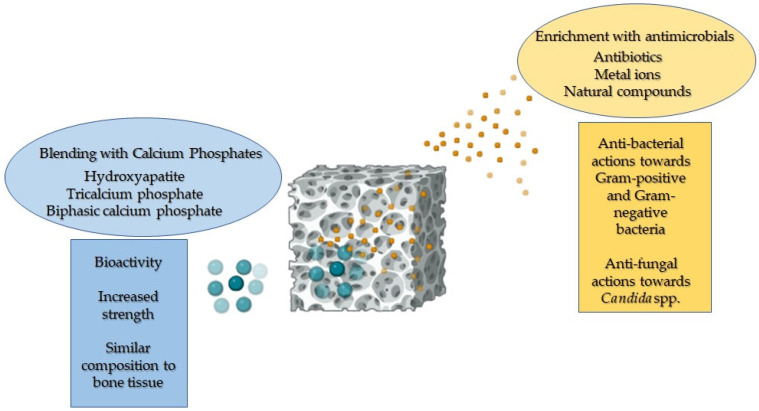
A representative image illustrating the enhanced properties of PCL when blended with calcium phosphates and functionalised with antimicrobial agents.

Moreover, PCL can be shaped into various forms using different techniques. It can be blended with calcium phosphates (CaPs) to promote bone deposition and provide adequate strength, and supplemented with antimicrobials to counteract implant-related infections without compromising its compatibility with human cells (Figure 2) [1,12,13]. Notably, several studies have demonstrated that enriching PCL with natural polymers, such as chitosan and gelatin, further enhances human cell adhesion and proliferation by providing cues for these cells. This increase is attributed to the enhancement in surface and bulk hydrophilicity [10,14,15,16].

Several review articles have focused on the use of PCL in tissue engineering; however, to the best of our knowledge, none have specifically addressed bone tissue engineering. This review article focused on the available and most recent literature regarding the application of PCL scaffolds in bone tissue engineering, encompassing their elaboration processes and modification with additives to enhance functionality. Initially, we explored both conventional and innovative techniques used to produce scaffolds for bone tissue engineering applications. Additionally, we described the use of various calcium phosphates to promote implant mineralisation and osteoblast colonisation. The incorporation of antimicrobial compounds, specifically antibiotics, metal ions, and natural extracts, was examined to assess advancements in the antibacterial and antifungal performance of PCL-based biomaterials. Subsequently, we discussed the literature on the cytocompatibility and promotion of human cell adhesion and proliferation on these constructs. Finally, we emphasised the challenges and future perspectives of PCL-based biomaterials.

## 2. Development Processes of PCL-Based Biomaterials

PCL and PCL-based scaffolds for bone tissue engineering can be fabricated either through conventional processes or innovative 3D printing techniques.

**Figure 3:**
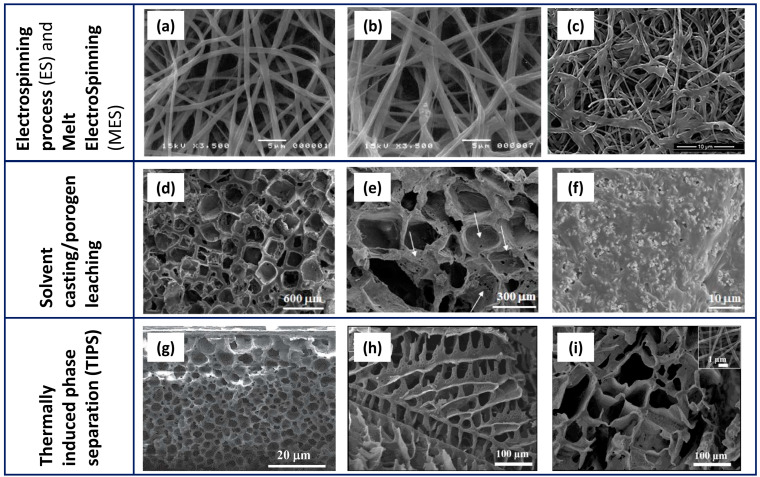
Representative micrographs of PCL-based scaffolds fabricated by ES/MES (**a**–**c**), SC/PL (**d**–**f**), and TIPS (**g**–**i**). Electrospun PCL (**a**), PCL with 1.0% HA (**b**) scaffolds (reprinted from [17] under the CCC license n. 5766001110392), and (**c**) Cu-doped wollastonite/PCL scaffolds after immersion in simulated body fluid for 14 days (reprinted from [30] under the CCC license n. 5766071417184). Lower (**d**) and higher (**e**) magnification images of PCL scaffolds and PCL/biphasic calcium phosphate scaffolds obtained by the SC/PL method, using NaCl as a template; (**f**) a biphasic calcium phosphate (BCP)/PCL sample showing the fine and homogeneous distribution of the calcium phosphate particles inside the polymer matrix (reprinted from [42] under an open access Creative Common CC BY license). TIPS-derived scaffolds containing PCL and virgin olive oil (**g**) (modified from [52] under the CCC license n. 5766100665534) and PLA/PCL/gelatin nanofibers (**h**) also added with 0.1% taurine (**i**) (reprinted from [53] under an open access Creative Common CC BY license).

Electrospinning, solvent casting/porogen leaching, and phase separation are the most widely used traditional fabrication methods. A brief description of these techniques is provided below, while Table 1 summarises the main features of the scaffolds, the advantages, and the open challenges for each technology. When possible, results achieved through the incorporation of osteoconductive particles (such as CaPs) and antimicrobial agents, during the same fabrication process, have been highlighted. Additionally, Figure 3 presents representative micrographs of PCL scaffolds fabricated using the main traditional techniques.

In the electrospinning process (ES) and melt electrospinning (MES), a continuous filament is drawn from a polymer solution (ES) or from a melt (MES) through a spinneret by high electrostatic forces and deposited on a conductive collector [17,18]. Scaffolds developed through these methods typically exhibit a high surface-area-to-volume ratio, and high porosity and fibre diameters ranging from nanometre to sub-micrometre scales [19]. The variation of specific parameters such as solution concentration, applied voltage, and distance between the injector tip and collector [20] enables the tuning of the scaffold characteristics, including fibre diameter, alignment, and micro/nanostructure [21].

PCL can be further blended with natural or synthetic hydrophilic polymers to improve biodegradability and achieve some degree of hydrophilicity [18]. Examples of electrospun fibrous scaffolds made from PCL blended with natural polymers (such as chitosan [22], silk, and gelatin [23]) or synthetic ones (such as PLGA and PLA) [24,25,26] have been successfully reported. Additionally, the incorporation of calcium carbonate or calcium phosphate particles into the polymer solution or melt has been shown to enhance osteoblast proliferation and differentiation [17,27].

The electrospinning process can be performed applying different equipment configurations. For instance, single and multi-channel ES can be used to feed different polymers (e.g., PCL and PLA) either from a mixed solution in the former case or separately in the latter, where the feeding configuration affects both mechanical properties and cell adhesion [26]. Furthermore, uniaxial and coaxial configurations can be used to prepare single-phase or core–shell fibres. Particularly, Prado-Prone et al. [25] developed electrospun PCL with antibacterial features by incorporating zinc oxide (ZnO) particles within the fibres [28]. They fabricated fibrous materials by electrospinning a ZnO-PCL solution/suspension using acetic acid as a green solvent. Two electrospinning layouts were tested: uniaxial fibres produced from ZnO-PCL solutions, and coaxial fibres fabricated with a PCL inner-core and ZnO-PCL outer shell. Antibacterial activity of both types of fibrous materials was tested against *Escherichia coli* and *Staphylococcus aureus*. Results revealed a significant boost in the antibacterial efficacy of mats through a coaxial-fibre configuration compared to traditional uniaxial ones. The mats effectively restrained the growth of both planktonic and biofilm-embedded bacteria, likely via two main antibacterial mechanisms: (1) the release of Zn^2+^ ions, primarily sourced from Zn acetate nanoparticles, and (2) the photocatalytic oxidative actions facilitated by ZnO nanoparticles. Similarly, antibacterial core–shell fibres were fabricated by co-axial electrospinning [29]. In this case, the core was composed of xylan (the major component of hemicellulose in plant cell walls, with immune regulatory, antioxidant, and anti-tumour properties) suspension, while the shell was composed of a PCL solution. Additionally, levofloxacin was dissolved in the xylan solution and demonstrated excellent bactericidal performance against *E. coli* and *S. aureus*. Moghaddasi et al. [25] prepared composite electrospun fibrous scaffolds made of PCL/PLA/HA; the mixture was dissolved and dispersed in a N,N-dimethylformamide (DMF) and dichloromethane (DCM) mixture, to which was added *Nigella sativa*-derived essential oil, prior to electrospinning. The research demonstrated the antibacterial properties against Gram-positive bacteria of these fibrous constructs, highlighting the need to tune the concentration of *Nigella sativa* (no higher than 15%) to avoid a cytotoxic effect against fibroblast cells. Abudhahir et al. [30] combined PCL with wollastonite and copper (Cu) ions using the electrospinning technique, to achieve both osteoinduction and antimicrobial behaviour. In particular, the presence of Cu improved the amount of adsorbed protein on the scaffold, facilitated apatite formation in vitro, and exhibited a potent antibacterial effect against *S. aureus* and *E. coli*, while preserving biocompatibility towards mouse mesenchymal stem cells [30]. To overcome the well-known issue of PCL hydrophobicity, a possible solution involves mixing it with natural or synthetic polymers, as previously mentioned. Although not the topic of this review, the use of alternative strategies are also discussed, precisely functionalisation [31] or coating [32] to improve hydrophilicity. For instance, Goreninskii et al. [32] developed electrospun PCL scaffolds, subsequently covered by a diamond-like layer under a nitrogen atmosphere, showing a clear decrease in the water contact angle without affecting cell viability.

The solvent casting/porogen leaching (SC/PL) technique consists of a two-step process. In the solvent casting stage, the polymer is dissolved into a suitable solvent, to which are added water-soluble porogen particles. The mixture is then poured into moulds of the desired shape and dried under various conditions to remove the solvent (e.g., air drying, vacuum drying, and freeze-drying). The dried samples are subsequently subjected to a porogen leaching step in water, which is then removed within a short timeframe (approximately 48 h). Both inorganic salts (e.g., sodium chloride (NaCl) and sodium bicarbonate) and natural/synthetic polymer particles (e.g., sucrose, fructose, polyvinyl alcohol (PVA), low-molecular-weight polyethylene glycol) can be used as porogens. This technique produces scaffolds with approximately 70–90% porosity, depending on the amount of added salt. High porosity levels are necessary to successfully reach the interconnection of pores. The diameter and morphology of pores strictly depend on the size and shape of the salt/polymer crystals, making this a very reproducible technique. The main disadvantage is related to the complete removal of the porogen, which poses a challenge, particularly in the case of large samples. Additionally, an open and fully interconnected porosity is needed to achieve full porogen removal. In the literature [33,34,35,36,37,38], many examples are available related to the fabrication of PCL/CaP composite scaffolds using the SC/PL technique and conversely, a very limited number of studies address the addition of antibacterial agents. A few previous studies have investigated the role of antimicrobial agents, such as copper oxide [39], ZnO [40], and β-silver vanadate oxide (AgVO)_3_ [41] particles, in non-porous solvent cast films. Nevertheless, the authors’ previous studies [42,43,44,45] focused on the fabrication of high-porous PCL and calcium phosphate–PCL 3D constructs made using the SC/PL technique. The results exhibited the possibility to impart antimicrobial properties to the scaffolds by adding silver particles or essential oils during their fabrication [42,43,44,45]. The data achieved using this strategy are thoroughly discussed in Section 4.

The thermally induced phase separation (TIPS) technique is based on mixing a polymer dissolved in organic solvents (e.g., dioxane, tetrahydrofuran, dimethylformamide) with water (nonsolvent), generating polymer-poor and polymer-rich phases. The latter is cast into moulds, frozen, and freeze-dried under vacuum to remove the frozen solvent, leading to the formation of pores [46]. The polymer concentration, solvent/nonsolvent ratio, and cooling rate are the major processing parameters that play a role in influencing the morphology of scaffolds [46,47]. In fact, an efficient control over the final structure of the scaffold in terms of the morphology, average size of pores, and degree of their interconnection can be achieved adjusting these parameters [46]. The addition of calcium phosphate particles influences the architecture and properties of scaffolds, leading to an increase in the elastic modulus, a decrease in porosity, and an improvement in osteoconductive properties [48,49]. This technique allows the fabrication of a nanofibrous structure, in which can be achieved an additional and larger porosity by adding leachable porogen to the mixture [50], similar to the SC/PL technique.

Mixtures of PCL with natural polymers are not easily fabricated [51,52], necessitating a combination with other techniques, such as electrospun gelatin fibres [53] or post-coating with chitosan or gelatin [54]. In a broader context, researchers have explored the combination of TIPS with various fabrication techniques, including electrospinning [55], porogen leaching [56], and 3D printing [57]. These advancements have facilitated the creation of diverse architectures and morphologies of pores at the micro/nanometre scale, tailored for specific applications. These innovations have yielded porous scaffolds with a range of structures, including micro/macroporous [56], fibrillar (nano/micro-fibrous) [46], isotropic (random pore) [46], and anisotropic (oriented/aligned pore or microtubular) [58].

Few previous studies have demonstrated the feasibility of using the TIPS technique to incorporate antimicrobial agents into the primary polymer solution. Farzamfar et al. [59] developed bone scaffolds made of PCL/PLA containing the antibiotic tetracycline hydrochloride, whose addition provided antibacterial and osteoinductive properties. Shu and colleagues [60] designed membranes composed of PCL/PLA/nano-HA, with the addition of a zeolite-imidazolate framework loaded with Cu, and demonstrated an antibacterial effect against *E. coli* and *S. aureus*.

**Table 1:** Three main traditional techniques to fabricate PCL-based scaffolds for bone tissue engineering.

Technology	Scaffold Main Features	Advantages	Open Challenges	References
ES and MES	▪ Densely packed fibrous structure ▪ Random orientation of the fibres ▪ High porosity ▪ High surface-to-volume ratio ▪ Fibres from submicrometric to nanometric size for ES ▪ Larger diameters of fibres (micrometric) using MES ▪ Low thickness of scaffolds (1 mm max)	▪ High control over geometrical features of scaffolds (e.g., hierarchy, fibre diameter, alignment) ▪ Possibility of blending with natural or synthetic polymers ▪ Possibility of adding inorganic fillers (e.g., calcium phosphates and carbonates) ▪ Possibility to match ES with other techniques: self-assembly ES, template-assisted ES, layer-by-layer ES	▪ Limitation in thickness (1 mm max): 2D scaffolds ▪ Small volumes of scaffolds at low rates ▪ Need of (toxic) solvents to dissolve the polymer ▪ For MES, worse hydrophilicity and minor bioactivity to support growth and adhesion of cells	[18,19,21,25,61,62,63]
SC/PL	▪ 3D scaffold ▪ High porosity (70–90%) with good degree of interconnection ▪ Tuneable porosity selecting the amount, size, and shape of porogen	▪ Easy technique ▪ No complex or expensive equipment ▪ Good reproducibility ▪ Possibility to load calcium phosphate and antimicrobial agents	▪ Small samples ▪ Possible retention of toxic solvents ▪ Risk of residual salts in the scaffolds ▪ Co-addition of natural polymers still not available in literature	[42,43,44,45,62,64]
TIPS and TIPS/PL	▪ Fibrous membranes or 3D scaffolds	▪ 3D fibrous scaffolds ▪ Hierarchical porosity by addition of pore former ▪ Tuneable scaffolds by adjusting processing parameters ▪ High versatility in the architecture of scaffolds ▪ Possibility to combine TIPS with other shaping methods	▪ Toxic solvents ▪ Sublimation of the frozen solvent is, relatively, a time- and energy-consuming process ▪ Mixture of PCL and natural polymers is not easily fabricated	[46,51]

Despite being commonly employed, traditional techniques display several drawbacks. Particularly, their inability to fabricate scaffolds beyond simple shapes, their lack of customisation to match defect characteristics of patients, and their incompletely controlled structure/microstructure. Various 3D printing techniques such as extrusion-based printing, inkjet printing, selective laser sintering, fused deposition modelling, and vat-photopolymerisation have been proven as effective in shaping complex tissue scaffolds tailored to desired specifications [65,66].

Scaffolds produced through 3D printing methods typically exhibit full interconnectivity, and their porosity can be readily adjusted through the optimisation of processing parameters. This technology offers a unique opportunity to investigate how micro-architecture influences the proliferation of cells and the generation of the extracellular matrix. Tissue geometry can be derived from computed tomography or magnetic resonance imaging scans of patients, and reconstructed into 3D models [67]. Additionally, computational tools can simulate biomechanical and transport properties, enabling the design of specific scaffold architectures to maximise tissue growth within a scaffold-guided environment [68].

Table 2 reports the most commonly used 3D printing technologies for PCL-based scaffolds, and provides a brief overview of the main features of these constructs, as well as their advantages and the open challenges. The recent advances in fabricating antimicrobial 3D-printed scaffolds have been highlighted, as it was previously performed with traditional processes.

Among the extrusion-based techniques, fused deposition modelling (FDM) is widely used for the development of scaffolds based on pure or functionalised PCL, especially due to low melting temperature of the polymer (>60 °C). Besides producing materials with high strength [72], the easiness, reliability, and cost-effectiveness of the process contribute to its success. In FDM, the thermoplastic filament is heated to its melting point and then extruded through the heated nozzle onto a platform, forming a 3D structure. The adhesion between successive layers is achieved through high-temperature deposition, which re-melts the surface of the printed part, enabling bonding with the subsequent layer. A further advantage of this technique is its solvent-free nature. The features of the printed parts can be tuned by changing printing parameters, such as printing speed and temperature [66].

Numerous studies proved the feasibility to add calcium phosphate particles into melts of PCL, and extrude composite filaments, to be further used in the 3D printing process [73,74,75,76,77,78,79,80]. Composite scaffolds made of PCL and HA at 20 wt% have been fabricated using either micro- or nano-sized HA particles, and 3D pore structures of different sizes were produced successfully [73]. Similarly, PCL/HA (up to 20%) composite filaments were used to fabricate scaffolds. HA played a role in enhancing the compressive strength and especially the Young’s modulus: the researchers determined an increase by 30% in the filaments and by 50% in the scaffolds [80]. Safiaghdam et al. [74] developed PCL/β-tricalcium phosphate (β-TCP) 3D constructs with and without magnesium oxide (MgO) nanoparticles, and proved a role of MgO in boosting the osteogenic capacity in vivo. To this aim, the inorganic fillers were added to melts of PCL, and filaments were used to print lattice scaffolds [74]. Analogously, Krobot et al. [75] prepared blends of poly(3-hydroxybutyrate)-(PHB)/PLA/PCL and incorporated TCP particles as a bioactive filler. Composite filaments were used to print simple lattice scaffolds [75]. Their mechanical properties were in the range of human trabecular bone, and they resulted in not being cytotoxic. Kim et al. [76] developed PCL/β-TCP composite scaffolds that were submitted to a surface amine plasma polymerisation. The surface treatment improved the hydrophilicity of the scaffold, as well as the ceramic particles enhancing the bioactivity of pre-osteoblasts, proving the promising properties of these scaffolds in bone tissue applications [76]. In a study, PCL/biphasic calcium phosphate (BCP) composite scaffolds were created and in vivo-implanted subcutaneously, proving to have both biocompatibility and osteoconductive properties [81]. Likewise, composite PCL/CaP scaffolds were designed as a honeycomb-like structure and showed a complete interconnectivity of the pores, as well as an improved biodegradability due to the presence of CaPs [79]. Human mesenchymal stem cells, seeded on the constructs, were able to adhere, migrate, and differentiate into the osteogenic lineage [79].

The feasibility to print blends of PCL with natural polymers was demonstrated by Duymaz et al. [82]. They 3D-printed mixtures of PCL, gelatin, and different concentrations of a low-molecular-weight polysaccharide (Halomonas levan, HLh). Therefore, cellular behaviour of human osteoblasts was studied, resulting in an increased biocompatibility proportionally to HLh content [82].

The low melting temperature of PCL allows the addition of (certain) drugs in the melt, without thermal degradation [66]. However, several limitations remain regarding heat-sensitive biomolecules, and the failure to seed cells. In this scenario, Kim et al. [83] demonstrated the feasibility to load stents with antibiotics (amoxicillin and cefotaxime), which were directly added to molten PCL. Stents showed good mechanical properties, and the efficacy of antibiotics was successfully retained after printing. Muwaffak et al. [84] proved the opportunity to load zinc, copper, and silver into melted PCL, to achieve their controlled release and to impart antimicrobial properties. Salmria et al. [85] also demonstrated the incorporation into PCL of an antibacterial substance, such as silver sulfadiazine, whereas Wang at al. [86] used Zn (1–3%)-loaded PCL melts to produce filaments, and to print lattice scaffolds. The addition of Zn improved their mechanical properties and promoted the formation of new bone tissue up to 8 weeks after in vivo implantation. Notably, the greatest osteogenic effect was underlined when Zn was at 2 wt%.

Selective laser sintering (SLS) is a further, well-exploited, 3D printing technique to fabricate PCL-based scaffolds. The computer-controlled laser beam sinters the powder; once sintered, a roller spreads a new layer of powder, while the remaining one provides a structural support [65]. A carbon dioxide laser is usually applied for PCL [65]. The key feature of the technique is the surface roughness of the printed parts. Han et al. [87] carried out systematic research on how parameters of the SLS process affect the surface roughness of PCL scaffolds and the relationship between roughness and biocompatibility of constructs. Scaffolds were fabricated using various laser powers and scanning speeds. Observations revealed that elevated energy density compromises shape fidelity concerning pore size and porosity, leading to dense and smooth scaffold surfaces featured by an inferior cytocompatibility. Conversely, lower energy density yielded to a diminished mechanical strength; thus, the resulting roughness of the surfaces, attributed to incomplete sintering of PCL particles, allowed cell adhesion and proliferation [87].

Borate bioactive glass (BBG)/PCL composites were developed by SLS, to exploit the excellent biodegradability and osteogenesis of BBG [88]; likewise, CaP/PCL composites were also fabricated through SLS [47,89,90,91]. Liu et al. [47], using SLS, developed PCL/HA scaffolds characterised by interconnected pores able to support the proliferation of cells and the penetration of blood vessels. Furthermore, the authors highlighted that the loading of vascular endothelial growth factor onto the scaffolds further enhanced angiogenesis and osteogenesis.

Concerning the fabrication of antimicrobial PCL-based scaffolds by SLS, to the best of the authors’ knowledge, no previous literature is available.

Vat-photopolymerisation techniques include Stereolithography (SLA) and Digital light processing (DLP). In these processes, a photocurable liquid monomer is polymerised layer by layer by an ultraviolet (UV) light, being the UV source, a laser, in the case of SLA and a projector in DLP. Among the mentioned 3D printing technologies, vat-photopolymerisation processes provide the highest printing precision and resolution, as well as the best control over scaffold inner geometries. The design and printing of complex shapes is well proved, with a careful control in terms of porosity amount, size, shape, and interconnectivity [92].

As a major concern, it was recently observed that UV light poses a significant risk to DNA cells, potentially leading to dermal cancer [19]. This problem can be solved by using visible light during SLA bioprinting [92]. In addition, as a key post-printing step, scaffolds need to be carefully washed, in order to remove the remaining photoinitiator and uncured resin, since the presence of moieties can induce cytotoxicity.

As a further challenge, PCL has been modified with acrylates, methacrylates, and fumarates, to allow photopolymerisation, but only a few studies have reported the use of photocrosslinkable PCL in SLA [93,94,95,96]. In particular, Elomaa et al. [96] used solvent-free SLA to prepare a photocurable PCL-based resin: the resulting scaffold had high accuracy, no shrinkage, and interconnected pores of suitable size and shape.

**Figure 4:**
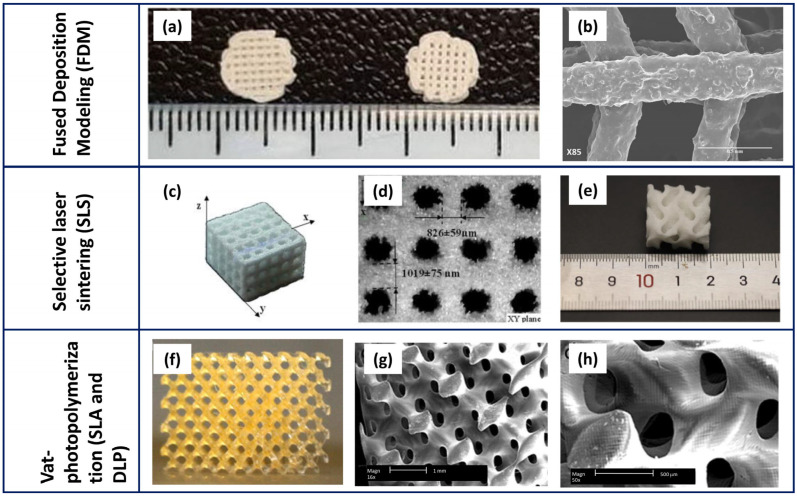
Representative micrographs of PCL-based scaffolds fabricated by 3D printing techniques: FDM (**a**,**b**), SLS (**c**–**e**), and SLA/DLP (**f**–**h**). (**a**) PCL/β-TCP (**left**) and PCL/β-TCP/nano-MgO (**right**) 3D-printed scaffolds, and (**b**) details of pore and strut size (modified by [74] under the CCC license n. 5766120164887); (**c**) PCL/HA (70:30) composite lattice scaffolds fabricated by SLS and (**d**) strut sizes measured in the different directions (modified by [90] under the CCC license n. 5766120654002); (**e**) irregular shape of a PCL/HA scaffold (modified by [91] under the open access Creative Common CC BY license); (**f**) a digital photo of a PCL scaffold fabricated by SLA and its related microstructure at lower (**g**) and higher (**h**) magnification (modified by [96] under the CCC license n. 5766540484597 and 5766550004916).

Recently, bioprinting has been gaining increasing interest. In fact, the seeding of cells on the scaffolds can result in a non-uniform distribution of cells, while decreasing its effectiveness. Bioprinting allows the deposition and uniform distribution of living cells, and other biomolecules, simultaneously to printing [97,98]. Bioprinters typically have several printing nozzles, one for printing polymers (e.g., PCL) and one for printing cells as well as heat-sensitive materials. This method allows the integration of various cells into scaffolds, thus promoting tissue formation [69,98]. Figure 4 depicts some representative SEM micrographs of PCL-based scaffolds fabricated using the main 3D printing techniques. If compared to Figure 3, showing traditional methods, the higher resolution and capability to control the geometrical features is evident in the scaffold. This is especially visible through the vat-photopolymerisation techniques that allow complex and unprecedented inner architectures.

## 3. Blending of PCL-Based Biomaterials with Calcium Phosphates to Allow Osteogenesis

Calcium phosphates (CaPs), the mineral elements of bone, can be used in combination with different materials, including metals, like titanium (preferentially as bioactive coating [99,100]), or with polymers. The latter provide composite mixtures to be used as injectable materials (such as acrylic cements) for bone repair and implant fixation [101,102], drug delivery systems [103,104], and scaffolds [105]. CaPs are mainly represented by hydroxyapatite (HA), β-tricalcium phosphate (β-TCP), and their mixture biphasic calcium phosphate (BCP). CaPs are featured by different properties, especially osteoinduction and osteoconduction, since they are similar in composition to bone. Osteoconduction refers to the ability of a biomaterial to serve as a scaffold for the growth of new bone tissue, meaning that a hierarchically porous structure is mandatory for this aim. Upon implantation, the biomaterial interacts with body fluids, resulting in the dissolution of calcium and phosphate ions from its surface. These ions reprecipitate on the scaffold surface, generating a biological apatite, whose chemical and structural similarity to natural bone promotes human cellular attachment and proliferation, thus initiating the bone remodelling process [106]. Conversely, osteoinduction refers to the ability to induce the differentiation of progenitor cells into osteoblasts, leading to the formation of new bone tissue. As the mechanism occurs through the release of bioactive factors (e.g., bone morphogenetic proteins, BMPs) that stimulate the recruitment and differentiation of cells, pure CaPs are typically not inherently osteoinductive. However, they can display this characteristic when combined with bioactive factors. The in vivo osteoinductive properties of CaP have already been reported in the literature, and associated with their high affinity for BMPs and growth factors. Therefore, CaP acts as a concentrator of osteoinductive molecules, also providing this feature to the ceramic material [107].

PCL is believed to be one of the key biomaterials for applications in bone tissue engineering, as it possesses outstanding characteristics such as biocompatibility and biodegradability. Literature reports show its use in repairing a small tract of bone defects to allow its healing. In addition, examples of osteoinductive electrospun PCL scaffolds have already been reported. In fact, the loading of this polymer with proper pro-angiogenic agents such as dexamethasone, simvastatin [108], and cholecalciferol [109] successfully conferred to PCL osteoinductive properties. It can also be blended with CaPs, being composite materials, enhancing the hole compressive strength and improving the rate of osteogenesis [110,111].

**Figure 5:**
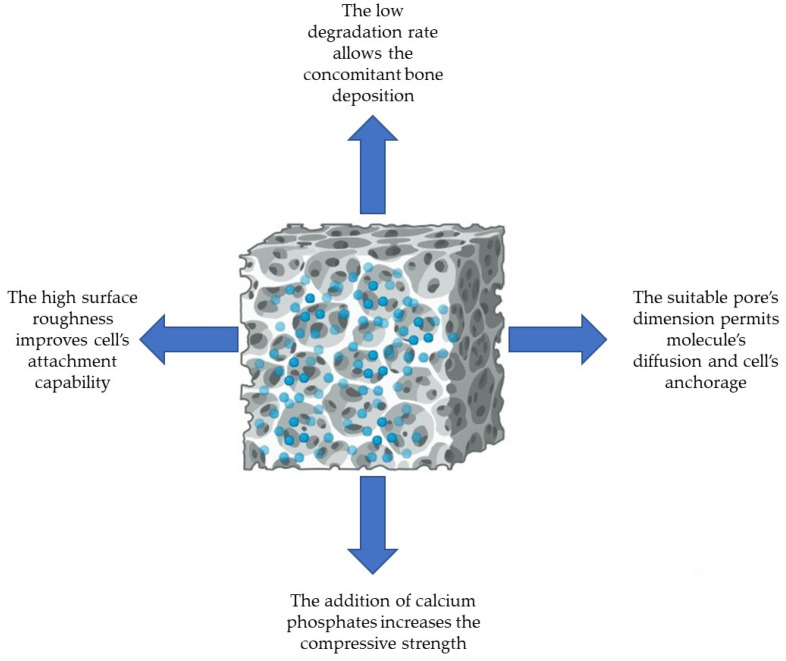
The relevant features of PCL-based 3D scaffolds blended with calcium phosphates designed for bone tissue engineering.

β-TCP was the first CaP used as a bone graft substitute, already in 1920. Notably, upon its utilisation, the resultant formed bone mass typically diminishes from its original quantity due to resorption. This is the reason why β-TCP needs to be used in association with another compound featured with a slow resorbable period [112]. Thereafter, in the 1970s, HA became available in this field, representing the predominant CaP component of bone. In an in vivo environment, these CaPs display different behaviour; in fact, HA, with its low solubility constant, is often regarded as non-resorbable, whereas β-TCP, being highly soluble, undergoes resorption [113]. β-TCP is also able to neutralise the acidification milieu that occurs during the degradation process of the polymers [114]. Therefore, their mixture that is BCP, at a different ratio, is an assessable solution since it combines the properties of both. Due to their similarity to bone and to their cytocompatibility, CaPs (e.g., HA, β-TCP, and BCP) are widely used in bone tissue engineering for orthopaedic applications. Moreover, the low degradation rate of PCL allows bone tissue regeneration during its degradation period. Figure 5 reports the key characteristics achieved by composite biomaterials based on PCL and CaPs.

Consequently, composite biomaterials based on PCL and CaPs are highly desired due to the properties achieved from their combination [115]. In a notable study, the researchers demonstrated that a composite multi-layer scaffold made of PCL and BCP is a suitable solution for bone tissue engineering since each layer confers additional properties to the construct: resistance in the bulk (ceramic core) and porosity in the surface (polymer external layer). Additionally, these 3D scaffolds displayed the appropriate gradient of porosity and degradation rate [113]. In a work of our research group, a functionally graded 3D scaffold was designed with a ceramic inner core and a PCL external layer. The characterisation performed with field emission scanning electron microscopy (FESEM) allowed us to demonstrate that the different layers were closely linked and the degradation rate test revealed the bioactivity of the inner core, thus being a promising construct for bone tissue applications [116]. More recently, Rezania et al. [80] obtained a scaffold made of PCL and HA, of which they observed the uniformity of pores and the dispersion of HA into the constructs, with agglomeration in some parts. Gerdes and colleagues [111] developed 3D-printed scaffolds based on PCL and enriched with HA, as composite material, and revealed close spaces within the pores. Additionally, when PCL and HA were blended in a 3D scaffold, a fine distribution of HA was revealed, with aggregates of different sizes [115]. In another study, 3D constructs made of PCL and blended with HA were characterised, and the presence of CaPs in their structure was highlighted [117]. More recently, PCL was successfully incorporated with 1% of HA into 3D scaffolds and their increase in roughness improved the performance of cell attachment [118]. Miszuk et al. [119] produced biphasic PCL and HA nanofibrous scaffolds by electrospinning with high interconnected pores, whose diameter was about 2.4 μm. PCL was enriched with HA and fluorapatite to fabricate 3D composite constructs, which were subsequently examined using scanning electron microscopy (SEM): the analysis revealed the presence of apatite particles in the surface [120].

In a recent study, the researchers developed a 3D functionally graded scaffold composed of PCL, gelatin, and nanohydroxyapatite. Each layer was interconnected to achieve a suitable porosity, and the construct displayed an initially high degradation rate, within the first two days, which subsequently decreased [121]. In a similar study, the researchers produced comparable scaffolds and reported both an average pore size of 4.7 ± 1.04 μm and a uniform and adequate deposition of nanohydroxyapatite on the surface. The degradation in an aqueous medium led to structural rupture of the scaffolds [4].

In another study, electrospun scaffolds composed of PCL, HA, and chitosan were prepared. An SEM analysis revealed the interconnected porosity of the specimens and the presence of HA. The bioactivity test highlighted the deposition of an apatite layer after 6 weeks of incubation [16]. Similarly, PCL and chitosan cubic-shaped scaffolds exhibited a squared porosity with an average width of 440 ± 16 μm and a height of 120 ± 5 μm [10].

PCL-based scaffolds produced using the melt electrowriting technique demonstrated an increased surface roughness when compared to those prepared with PLA or with PLA/PCL [122].

Cylindrical-shaped granular multichannel bone substitutes were developed using BCP (60 HA + 40 β-TCP), and the internal porosity and compressive strength were assessed. The results revealed a high bulk macro-porosity with pore diameters of 1, 2, and 3 mm, and that the compressive strength increased proportionally with the diameter of pores [2].

Thuaksuban et al. [123] produced three types of 3D scaffolds: pure PCL, and PCL incorporated with BCP at 20% or 30%—they demonstrated that the scaffolds had large pores and released calcium and phosphates over time. However, when the BCP content was increased to 30%, fractures were observed within the constructs. More recently, our interdisciplinary group designed PCL-based scaffolds blended with BCP (70 HA + 30 β-TCP) and evaluated their physico-chemical properties from various perspectives. The results demonstrated that the 3D constructs were featured by a highly interconnected and homogeneously distributed porosity, due to the salt-leaching process [42]. When NaCl was used as pore-formed salt, the resulting pores displayed a regular squared geometry, whereas the use of sodium nitrate (NaNO_3_) produced pores with less defined geometry. Furthermore, the dimension of pores varied from approximately 234 µm with NaCl to 208 µm with NaNO_3_ [43]. Additionally, the FESEM analysis revealed the homogeneous and fine distribution of BCP within the porous 3D constructs, which also increased their stiffness compared to those made of pure PCL [42]. The degradation rate tests, conducted by immerging the constructs, for different incubation times, in simulated body fluids, revealed that pure PCL specimens experienced slow weight loss during 20 days of immersion. In contrast, the addition of BCP into the polymer determined a faster weight loss [43,44]. Kim and colleagues [76] fabricated PCL scaffolds with a plasma polymerisation for β-TCP deposition. An FESEM analysis demonstrated both the random distribution of β-TCP nanoparticles on the surface and the pores of ~300 μm, whereas the X-ray diffraction (XRD) analysis revealed peaks corresponding to PCL and β-TCP. In a study, 3D scaffolds were fabricated using PCL and 45 wt% of β-TCP, with or without the addition of 10 wt% of MgO nanoparticles. The resulting scaffolds exhibited a well-defined microstructure, a size of pores of about 500 μm, and an effective dispersion of MgO nanoparticles [74].

In another study, the researchers hybridised PCL with the copolymer Inulin-g-poly(D,L)lactide, resulting in scaffolds with squared pores, high surface roughness, and low degradation rates after 180 days of incubation [124].

Several papers reported the loading of CaPs and antimicrobial agents on 3D scaffolds based on PCL, and revealed no alterations in their structural integrity and interconnected porosity, while demonstrating sustained release of the incorporated compounds [125,126]. Conversely, a 3D scaffold based on PCL, PLA, and HA was enriched with *Nigella sativa*, known as black curcumin: the addition of the natural compound determined an altered morphology compared to the pure one [25].

Table 3 reports the key results of the literature analysis on the morphological and/or chemical characterisation of the PCL-based scaffolds blended with CaPs.

## 4. Antimicrobial Properties of PCL-Based Biomaterials Loaded with Antimicrobial Agents

PCL-based biomaterials for bone tissue engineering can be added with different antimicrobial agents to prevent bacterial and yeast adhesion, and biofilm formation on the device. The antimicrobials can be released by the construct and act towards free-floating microorganisms. The available literature data pertain to the enrichment of PCL with antibiotics or antifungals, metal ions, and natural compounds (e.g., essential oils), and the investigation of the antimicrobial properties by means of different approaches. Particularly, the release of antimicrobial compounds has been evaluated using an agar diffusion method comparable to the Kirby–Bauer test, while microbial adhesion has been quantified through experiments preceded by sonication. Additionally, FESEM has been employed to observe the inhibition of biofilm formation stages.

Only a limited number of articles reported the loading of antimicrobial drugs on 3D scaffolds based on PCL, designed for bone tissue engineering, aimed at evaluating their antibacterial properties. In these cases, the antibacterial features were conferred by the selective toxicity of the drugs depending on their mechanism of action. Specifically concerning antibiotics, PCL constructs were supplemented with doxycycline and the agar diffusion assay was employed towards *E. coli* K-12: the results demonstrated the elution of the drug and an inhibition halo of 10 mm [127]. Tetracycline was incorporated in composite scaffolds based on PCL and HA. The researchers observed, through an agar diffusion test, no inhibition halo on pure PCL, but a significant growth inhibition of two bacterial strains upon drug loading, with a larger inhibition halo measured for *S. aureus* compared to *E.coli* [125]. In a more recent study, tetracycline was blended in PCL and PLA porous membranes, which were evaluated for antimicrobial properties towards *S. aureus* and *E. coli* through an inhibition halo assay. The results confirmed the sustained antibacterial activity, up to 21 days, with a more pronounced action towards *E. coli* [128]. Similarly, PCL scaffolds were coated with PLA vancomycin-loaded microspheres, and showed a relevant anti-*S. aureus* action, observed over 28 days of incubation [129]. In a study, researchers introduced erythromycin in a coaxial structure based on PCL/PLGA-PVA. An agar diffusion test on *S. aureus* growth revealed that the inhibition halo diameter increased proportionally with the erythromycin concentration into the constructs (ranging from 10 to 1000 μg/mL) [130]. Another study involved the fabrication of scaffolds based on PCL and TCP loaded with ceftriaxone microspheres to evaluate the antibacterial activity against *E. coli*, resulting in an inhibition halo of 30 mm [131].

Due to the global rising problem of antimicrobial resistance to both antibiotics and antifungals, researchers draw their efforts towards elucidating alternative compounds, mainly metal ions. The latter offer the advantage of exerting multitargeted actions, which can result in a reduced likelihood of resistance induction compared to conventional antimicrobial agents [6,132]. Notably, metal ions can interact with key functions and/or structures of microorganisms, precisely targeting and disrupting their cell wall or cytoplasmic membrane, interfering, at various extents, with the metabolism of proteins, enzymes, and DNA. Additionally, they inhibit the biofilm formation of both bacteria and fungi [43,44,45].

In this scenario, silver emerges as a key additive to PCL to impart both antibacterial and antifungal activity. Afghah and colleagues [133] produced highly porous 3D scaffolds based on PCL loaded with silver, demonstrating varying degrees of antimicrobial efficacy against different pathogens. *Candida albicans* was the most susceptible microorganism (inhibition halo of about 16–17 mm), followed by *E. coli* and *S. aureus* (inhibition halo of about 9–10 mm), whereas *Pseudomonas aeruginosa* demonstrated the lowest susceptibility (inhibition halo of about 8 mm).

PLGA and PCL scaffolds were added in situ with silver nanoparticles and the agar diffusion tests showed antibacterial action towards both *S. aureus* and *Streptococcus mutans*, with inhibition zone diameters of ~13.5 mm and ~9.0 mm, respectively. Moreover, FESEM images revealed the bacterial attachment to the constructs [134]. In a study conducted by our research group, 3D composite scaffolds were enriched with silver at a concentration of 1.67%. While an *S. aureus* inhibition in growth and adhesion was revealed, this percentage impaired eukaryotic cells’ viability [42]. Therefore, silver concentration was reduced at approximately 1%, which exhibited no adverse effect on human osteosarcoma (Saos-2) cells, as well as an important antibacterial and antifungal activity. The inhibition halo experiments demonstrated the silver release from the constructs, leading to halted growth of all tested microorganisms, precisely *S. aureus*, *S. epidermidis*, *E. coli*, *C. albicans*, and *C. auris*. Additionally, the adhesion of bacteria and yeasts was reduced on the constructs added with silver, and planktonic growth of these microorganisms was also inhibited. Ultimately, we also demonstrated that only the 3D scaffolds enriched with silver showed no biofilm formation as confirmed by the FESEM analysis, which also revealed alterations in the usual morphology of bacteria and yeasts due to silver direct action on these cells [44,45].

PCL and HA constructs were loaded with different percentages of ZnO, and upon contact of *S. aureus* with these scaffolds, ZnO release determined a significant reduction in viable bacteria, mainly after 30 days of incubation. The most pronounced effect was observed at a concentration of 6% [126]. In another study, the researchers produced composite 3D membranes made of PCL blended with ZnO at increasing percentages, ranging from 1% to 7%. They demonstrated an effective antibacterial activity on *S. aureus* and *E. coli*, with a notable reduction in their adhesion to the constructs especially at 7% of ZnO [40]. Moreover, ZnO nanoparticles (0.5 wt% or 5 wt%) were added to PCL to hamper the growth of *S. mutans* and *Porphyromonas gingivalis*: no significant differences were obtained in the adhesion of these bacteria to the modified constructs compared to pure PCL [135].

The Abudhahir research group [30] developed PCL scaffolds reinforced with copper, and revealed a zone of inhibition measuring approximately 6.2 mm and 5.8 mm against *S. aureus* and *E. coli*, respectively. The greater activity towards *S. aureus* was attributed by the researchers to the intrinsic antibacterial properties of copper.

The research also exploited the effect of natural compounds added to PCL to improve the antimicrobial properties while avoiding the development of a resistant profile. In fact, the multitargeted action of these molecules hampers bacteria and/or fungi to become resistant, and the suitable concentration should be added in order to not impair the viability of eukaryotic cells [136,137,138,139,140]. Despite that the mechanism of action of essential oils (EOs) has not been fully elucidated yet, several studies have hypothesised the disruption of the microbial cell membrane, causing an increase in the permeability, the release of intracytoplasmic material, and an interference with cell metabolism and enzyme performance [43,141]. In a study, PCL and gelatin scaffolds were enriched with chrysin (at 5%), a natural flavonoid, and the antibacterial action was evaluated by both agar diffusion and live/death assays. The chrysin-enriched scaffolds were able to counteract *Acinetobacter baumannii*, *Ps. aeruginosa*, *S. aureus*, and *Enterococcus faecalis* growth due to the chrysin presence with an inhibition zone ranging from 8 mm to 12 mm, and additionally bacterial cells did not survive only in the enriched scaffolds [142]. Polo’s research group [143] produced scaffolds based on CaPs supplemented with vanillin, demonstrating an anti-*E. coli* effect only in the presence of this compound compared to the free-vanillin control. Additionally, an FESEM analysis revealed an altered bacterial morphology [143]. PCL scaffolds were added with increasing concentrations—0%, 2%, 4%, and 8%—of clove and red thyme to impart anti-*Candida tropicalis* biofilm production activity. The results demonstrated that *C. tropicalis* clinical strains were inhibited in biofilm formation when the essential oil concentration was at 4% [144]. More recently, PCL was blended with varying cinnamon and eugenol concentrations ranging from 30% to 50%. The resulting scaffolds demonstrated an antibacterial and anti-biofilm activity towards *S. aureus*, *S. epidermidis*, and *E. coli* as evinced by inhibition halo assays, adhesion experiments, and FESEM observations. The bacteria were also altered in their usual morphology with *S. aureus* exhibiting an enlarged shape and *E.coli* displaying filamentous forms [43]. Likewise, a 3D scaffold based on PCL/PLA and enriched with HA and *Nigella sativa* oil at concentrations of 15, 18, and 20 wt% was evaluated for the antibacterial properties against *S. aureus* and *E. coli* by the inhibition halo test. The data revealed that, when the natural compound was added, the antibacterial activity was obtained only towards *S. aureus* since *E. coli* displayed a natural resistance to *Nigella sativa* [25].

PCL was also loaded with graphene oxide (GO), and evaluated for its antibacterial efficacy on *S. epidermidis* and *E. coli*. Both bacterial strains were found on the scaffolds but, compared to pure PCL, an increased number of dead cells was observed, demonstrating a bactericidal action, more pronounced when the 7.5% of GO was introduced [8]. PCL was loaded with chitosan to obtain antimicrobial and anti-biofilm 3D scaffolds, which were evaluated for their effects on *S. aureus* and *S. epidermidis* growth after 24 h of incubation. Despite the chitosan molecular weight, a significant reduction in adhesion and biofilm formation for both bacteria was demonstrated compared to pure PCL constructs [10].

Table 4 reports a summary of the literature results pertaining to the antimicrobial features of the PCL-based scaffolds enriched with antimicrobials.

## 5. Cytocompatibility of PCL-Based Biomaterials Functionalised with Both Antimicrobials and Calcium Phosphates

Several types of eukaryotic cells can be used in bone tissue engineering applications to evaluate the cytocompatibility of 3D scaffolds. The most common eukaryotic cells pertain to mesenchymal stem cells, osteoblasts, and fibroblasts from different cell lines. These cells demonstrate robust attachment and proliferation on porous constructs with a pore size of about 200–400 μm [145]. The size of pores does influence cell penetration into a 3D scaffold: pores that are too small may prevent cell infiltration into the construct, leading to cellular aggregation outside it, whereas excessively large pores may limit cell bonding due to reduced surface area for attachment. Therefore, a good balance should be reached to permit cell adhesion, proliferation, and integration within the 3D scaffold [146]. Mesenchymal stem cells play a crucial role in bone regeneration, since they differentiate into osteoblasts under a suitable condition with soluble factors, and also modulate the formation of stroma during new tissue deposition [147]. Human fibroblasts, under appropriate environmental cues, might differentiate into osteoblasts, thus serving as a potential source of osteogenic cells [148].

**Figure 6:**
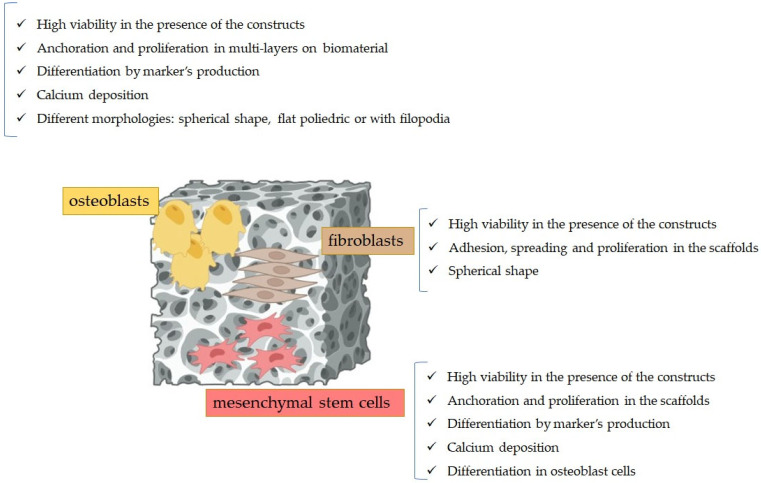
Results of the cytocompatibility experiments of the PCL-based scaffolds when posed in contact with eukaryotic cells.

Figure 6 depicts the most relevant results pertaining to cytocompatibility of PCL-based biomaterials on human mesenchymal stem cells, human fibroblasts, and osteoblasts.

Hejazi et al. [121] demonstrated that in the presence of 3D-graded scaffolds based on PCL, CaPs, and gelatin, mesenchymal stem cells were not impaired in their viability and proliferations, during the 14 days of incubations, since no toxic products were released by the constructs. Human mesenchymal stem cells were tested to determine the osteoconductivity of PCL-based constructs enriched with HA and ZnO (1% *w*/*w*). The immunostaining results revealed the expression of osteodifferentiation markers by these cells, along with significant calcium deposition, mainly in the HA presence. Additionally, the cells successfully colonised the scaffold and underwent differentiation into osteoblasts [149]. Previously, the same researchers demonstrated by an SEM analysis the presence and proliferation of mesenchymal stem cells on the same scaffolds produced without the addition of ZnO [111].

In a study, scientists blended PCL with silica microcapsules and revealed that mesenchymal stem cells were able not only to survive in the constructs but also to adhere and proliferate. A significant difference was observed between PCL decorated with silica microcapsules and pure PCL, as the latter exhibited minor surface roughness, potentially affecting attachment and the subsequent proliferation of mesenchymal stem cells [147].

Recently, the Malysheva research group [120] evaluated the cytocompatibility of 3D composite constructs produced with PCL functionalised with different concentrations of HA. Their data revealed that human mesenchymal stem cells were not hampered in their viability after 72 h of incubation, but only when HA was lesser than 7%, it promoted a greater attachment of human cells. They also performed differentiation assays on mouse mesenchymal stem cells (C2C12), revealing a higher early-stage differentiation marker production on PCL-HA than on pure PCL constructs [120]. Two types of 3D scaffolds were designed and assayed for cytocompatibility towards bone marrow mesenchymal stromal cells, specifically PCL with β-TCP (45 wt%) and PCL with β-TCP (45 wt%) supplemented with nano-MgO (10 wt%). The experiments revealed the osteoinduction of cells, a long-term viability when MgO was introduced, and an increase in alkaline phosphatase (ALP) activity [74].

Murine fibroblasts (L929) were used to evaluate the cytocompatibility of PCL scaffolds produced with the electrospinning technique by a 3-(4,5-dimethylthiazol-2-yl)-2,5-diphenyltetrazolium bromide) tetrazolium (MTT) assay. The data demonstrated that the cells were not impaired in their viability and not altered in their morphology, since they preserved the spherical shape [122]. The hybridisation of PCL with the copolymer Inulin-g-poly(D,L)lactide was investigated for its non-toxic behaviour towards human fibroblasts (MRC-5-CCL-171, American Type Culture Collection, ATCC^®^) and human adipose-derived mesenchymal stem cells. The results proved that at direct contact, fibroblasts displayed an adequate cytocompatibility, and the experiments on human adipose-derived mesenchymal stem cells confirmed a 100% viability, an enhanced attachment, and high cell density on the scaffolds, as well as an increased expression of differentiation markers [124].

PCL scaffolds, coated with chitosan, gelatin, and bioactive glass, were assayed for biocompatibility towards fibroblast cells (MG-63): the experiments revealed that human cells were not affected in viability and proliferation into the constructs, whereas the coating further enhanced the calcium deposition of cells [54]. Three-dimensional PCL/PLA constructs enriched with gelatin and taurine were evaluated for their toxicity on fibroblasts (MG-63) after 24 and 72 h after seeding: the results showed no compromise in the viability and proliferation of these cells [53]. Human fibroblast cells (CCD-1072-SK) were posed in contact with 3D scaffolds based on PCL/PLA and HA, enriched with black curcumin EO at increasing concentrations. The researchers demonstrated that after 24 h, a relevant number of viable cells was registered on both pure and EO-enriched constructs, whereas after 48 h, the number of viable cells further decreased respective to pure constructs [25].

Previously, research on osteoblast cells (MC3T3-E1, subclone 4) aimed to evaluate their anchorage, proliferation, and differentiation on pure PCL or PCL blended with BCP (at 20% or 30%) demonstrated that eukaryotic cells were not only able to attach and proliferate with multi-layers on the scaffold but also to differentiate as evinced by increased ALP activity and osteocalcin (OCN) gene expression. These results significantly differed from those observed with pure PCL scaffolds [123]. The same cells were investigated for their proliferation on PLA and PCL composite scaffolds. The researchers demonstrated that the scaffolds displayed a good cytocompatibility while allowing the proliferation of osteoblasts [47].

Recently, our research group designed 3D scaffolds made of PCL and blended with BCP, produced by the salt-leaching technique. These constructs were tested for their biocompatibility with SaoS-2 cells by an MTT assay, for their cell adhesion and proliferation via an FESEM analysis, and for calcium deposition by alizarin red S staining. The results demonstrated that the specimens were not toxic for osteoblast-like cells at different incubation times, and the addition of BCP did not impair their viability. Conversely, only low concentrations of either silver (~1%) or EOs (30%) did not reduce the viability and proliferation of osteoblasts [42,43,44]. The FESEM micrographs showed that the cells adhered and proliferated into the constructs, in particular after 7 days of incubation. The osteoblasts did not alter their morphology when posed in contact with the specimens, but they exhibited an elongated shape capable of a better anchoring to the constructs [42,43,44]. Additionally, we revealed that scaffolds were able to promote calcium deposition by SaoS-2 cells [45]. In accordance with our data, a study by the Rezania group [80] investigated the seeding of human osteoblast cells (MG-63) onto scaffolds composed of PCL and HA at varying percentages. The MTT assay demonstrated a non-toxic behaviour of the biomaterials, at 7 and 14 days. SEM images showed the colonisation of the constructs by the cells, which also maintained their spherical shape. Furthermore, alizarin red S staining revealed the calcium deposition of osteoblasts, further demonstrating their differentiation [80]. Similarly, when human osteoblasts were seeded on 3D scaffolds made of PCL and HA—at increasing percentages—these were shown to be non-toxic, as proved by the coverage of their surface by the cells, which properly proliferated and attached. The most suitable HA concentration was found to be 10% that allowed the spread of osteoblasts and the favourable flat polyhedral morphology [115]. In another study, 3D scaffolds based on PCL and blended with HA were assayed for their biocompatibility towards human osteoblasts (MG-63). The results revealed that the constructs were able to support cell proliferation, calcium deposition, and the upregulation of genes involved in differentiation. These results were more pronounced in PCL blended with HA compared to the pure one [117].

Recently, human osteoblasts (HOB-Promocell C-12720) were tested for adhesion and proliferation on 3D constructs made of PCL blended with 1% of HA: the cells increased their viability and proliferation along with enhanced calcium deposition compared to neat PCL. Moreover, an SEM analysis demonstrated the presence of osteoblasts with filopodia [118]. Osteoblast cells were also assayed for viability, proliferation, and morphological characterisation on scaffolds composed of PCL, gelatin, and nanohydroxyapatite. The experiments revealed an enhanced cell viability and proliferation of cells, which also displayed a polygonal-shaped morphology able to better colonise the 3D specimens. This colonisation was attributed to the rise in surface roughness determined by HA addition [4].

Pre-osteoblasts (MC3T3-E1) were tested for their viability, proliferation, and differentiation on PCL-based scaffolds enriched with β-TCP nanoparticles. The results demonstrated a high viability, proliferation, and adhesion of eukaryotic cells on the constructs as well as an increase in their ALP activity and mineralisation after 7 and 14 days of incubation [76]. Composite scaffolds made of PCL and Zn at increasing percentages (1, 2, or 3 wt%) were designed, on which MC3T3-E1 cells were seeded: the live/dead assay revealed no toxicity mainly when Zn content was at 2 or 3 wt% [86].

Finally, when PCL was blended with HA or TCP, and further enriched with antimicrobial agents such as antibiotics, metal ions, or essential oils, different results were obtained depending on the amount of these compounds [30,125,126,129,130,131,133,134,135,140,143]. For instance, Tamjid and colleagues [125] produced composite scaffolds made of PCL containing different concentrations of tetracycline hydrochloride and revealed that fibroblast cells displayed a higher viability pattern at a drug concentration of 1.15 mg/mL compared to 0.57 mg/mL. Conversely, another study revealed that human cells, specifically osteoblasts, when in contact with PLGA/PCL scaffolds in situ added with silver nanoparticles, exhibited the highest proliferative capability when silver was at the lowest concentration. The mineralisation of these cells was also highlighted, as well as their attachment to the scaffolds through filopodia [134].

Notably, chitosan and GO were selected to impart an antimicrobial effect on PCL-based constructs without affecting eukaryotic cells’ viability. PCL, HA, and chitosan were exploited to produce electrospun composite scaffolds and were posed in contact with human osteosarcoma cells (MG-63). The results demonstrated not only that these cells were viable within the constructs but also that an increase in HA concentration permitted a greater osteoblast proliferation. The researchers also demonstrated an upregulation of different genes involved in osteogenesis [16]. Cubic-shaped PCL and chitosan 3D scaffolds were tested for their cytocompatibility for adriamycin-resistant mouse fibroblast cells (L929). It follows that the constructs resulted in a high cell viability after 24 h and 7 days of incubation, and no changes in their phenotype were revealed [10]. Recently, PCL with chitosan was enriched with Zn at increasing concentrations (from 0 to 50) and the experiments on mouse fibroblast cells (NIH-3T3) demonstrated that only lower (10 and 20) Zn concentrations were not toxic for these cells, and allowed their attachment and proliferation into the 3D constructs [150]. A direct contact assay of human foreskin fibroblasts (HFF-1) on PCL fibrous scaffolds enriched with different percentages of GO demonstrated an irregular porosity proportional to the increase in GO content and that the scaffolds were able to promote the adhesion and spreading of eukaryotic cells, for up to 14 days of incubation [8]. Additionally, in another study, the researchers demonstrated that the GO presence (1% or 2%) in PCL-based scaffolds permitted human cell adhesion and proliferation even though at a lower rate compared to pure PCL [15].

Immortalised myoblast cells (C2C12) were posed in contact with biphasic PCL/HA nanofibrous scaffolds. The results demonstrated that cells attached and spread successfully into the construct, while significantly increasing their ALP activity, particularly when HA was added to the polymer [119].

Table 5 reports the key results of the literature analysis on the biocompatibility of PCL-based scaffolds.

## 6. Conclusions

The development of bone tissue engineered 3D scaffolds is an advanced approach to restore or even substitute damaged bone tissue, avoiding some serious disadvantages of autograft, allograft, and xenograft substitutes. Therefore, synthetic biopolymers, such as PCL, have become potential candidates to design constructs with favourable properties, mainly biocompatibility and biodegradability, associated with low costs and ease of shaping. PCL can be moulded into 3D scaffolds by using different elaboration processes and can be blended with calcium phosphates to enhance its stiffness and the potential of osteogenesis. Intriguingly, the slow rate of PCL degradation—combined with a suitable macro- and microporosity—allows the adhesion and integration of human cells into the construct. In fact, the pores inside the 3D scaffold can favourably permit the attachment, proliferation, and spreading of different cells involved in bone tissue healing. Additionally, the presence of HA and/or β-TCP further improves the cytocompatibility, osteoinduction, and mineralisation properties. Notably, several traditional and unconventional antimicrobial agents can be used to functionalise the 3D constructs to achieve anti-adhesive and anti-biofilm characteristics towards bacteria and fungi that might colonise the scaffold, thus diminishing its longevity upon implantation. The tuning of these compounds is necessary, since high doses can enhance microbial killing while impairing the viability of eukaryotic cells. Among the antimicrobial agents, not only antibiotics and antifungals can be employed, but also metal ions and natural compounds. The latter two did not display the drawbacks of the microbial resistance due to the multitargeted mechanisms of action. Our recently achieved results demonstrated that PCL-based 3D scaffolds with biphasic calcium phosphates displayed a high interconnected inner porosity that allowed eukaryotic cell attachment and proliferation. Notably, the addition of either silver or essential oils—at lower concentrations—was able to counteract bacterial and fungal growth, as well as their biofilm formation, by acting directly on these cells and altering their usual morphology. The progress in the design, manufacturing, and functionalisation achieved to date demonstrated in vitro the effectiveness of composite PCL/calcium phosphate 3D scaffolds for bone tissue engineering in the targeted healing of damaged bone. However, the step forward in this challenging scenario will be in vivo investigations in large animal models and in human clinical trials to further revolutionise and validate the efficacy and safety of these scaffolds in this constantly evolving biomedical field.

## Figures and Tables

**Table 2:** Three main 3D printing techniques to fabricate PCL-based scaffolds for bone tissue engineering.

Technology	Scaffold Main Features	Advantages	Open Challenges	References
Extrusion-based printing (EBP): FDM	▪ Simple lattice structures ▪ Small scaffolds: dimension of hundred microns, sometimes up to millimetres	▪ No solvent ▪ Continuous process ▪ High-strength materials ▪ Possibility of loading drugs and other agents ▪ Possibility of loading with natural polymer in the melt ▪ Possibility of loading CaP particles	▪ Low resolution ▪ The efficiency could be increased by Melt Electrospinning Writing (MEW) to print scaffolds based on micro-fibres ▪ The need of filament reduces batch dimension ▪ Risk of pollution ▪ Limited printing size and volumes ▪ No cell incorporation	[62,65,69]
Selective laser sintering (SLS)	▪ Simple and complex structures ▪ Porosity up 85% ▪ Hierarchical structure, with millimetre-sized pores and diffused microporosity (40–100 μm)	▪ High resolution ▪ Fast process ▪ Rough surfaces potentially improve adhesion of cells ▪ No solvent ▪ Possible multiple materials ▪ No filament needed ▪ Mechanical properties similar to trabecular bone	▪ Possible thermal damage ▪ Loading with antimicrobials not yet reported in literature ▪ High amount of waste due to high temperature ▪ The incorporation of cells and biomolecules is limited	[62,67,70,71]
Vat-photopolymerisation (SLA, DLP)	▪ Complex and unprecedented structures	▪ High accuracy ▪ High resolution	▪ Only photocurable polymers (PCL should be modified with acrylates, methacrylates, or fumarates to enable photo-polymerisation) ▪ Expensive equipment ▪ Cytotoxicity caused by photoinitiator or unreached monomers	[70]

**Table 3:** Summary of the key literature findings pertaining to morphological and/or chemical characteristics of PCL-based 3D scaffolds blended with calcium phosphates for bone tissue engineering.

Scaffold Composition	Key Findings	References
Composite multi-layer scaffold made of PCL and BCP	It had a resistant bulk (ceramic core) and a porous surface (polymer external layer), and displayed the appropriate gradient of porosity and a degradation rate	[113]
Functionally graded 3D scaffold designed with a ceramic inner core and a PCL external layer	The different layers were closely linked and the degradation rate of the inner core revealed bioactivity	[116]
Scaffold based on PCL and HA	The pores were uniformly distributed and HA was dispersed in the constructs, with some agglomeration	[80]
3D-printed scaffold made of PCL and enriched with HA	The composite material revealed small spaces among pores	[111]
PCL and HA blended in a 3D scaffold	HA in PCL-based scaffold was finely distributed with aggregates of different size	[115]
3D scaffold based on PCL and blended with HA	The appearance of CaPs was revealed in the structure	[117]
PCL incorporated with 1% of HA into 3D scaffold	The addition of HA to PCL provoked an increase in roughness	[118]
Biphasic PCL/HA nanofibrous scaffold	The pores displayed a high degree of interconnection. Their diameter was about 2.4 μm	[119]
PCL enriched with HA and fluorapatite to obtain a 3D composite scaffold	The presence of apatite particles was detected in the surface	[120]
3D functionally graded scaffold based on PCL, gelatin, and nanohydroxyapatite	The layers were interconnected to reach a suitable porosity. An initial high degradation rate within 2 days was recorded that, thereafter, slowed	[121]
3D functionally graded scaffold based on PCL, gelatin, and nanohydroxyapatite	It had an average pore size of 4.7 ± 1.04 μm with the uniform, and adequate deposition of nanohydroxyapatite on its surface, but the degradation in aqueous medium determined a rupture of its structure	[4]
Scaffold prepared with PCL, HA, and chitosan	Specimen with an interconnected porosity and with the presence of HA. The deposition of an apatite layer was highlighted	[16]
PCL and chitosan cubic-shaped scaffold	The construct presented a squared porosity (average width of 440 ± 16 μm and a height of 120 ± 5 μm)	[10]
PCL-based scaffold with or without PLA	An increase in surface roughness was observed in pure PCL scaffold compared to that prepared with PLA or with PLA/PCL	[122]
Cylindrical-shaped multichannel bone substitutes prepared using BCP (60 HA + 40 β-TCP)	A high bulk macro-porosity was revealed (1, 2, and 3 mm of diameter) and the compressive strength increased with the pore’s diameter	[2]
Three types of 3D scaffolds: pure PCL, and PCL added with BCP at 20% or 30%	The scaffolds had large pore size and released calcium and phosphates over time. BCP at 30% produced fractures in the construct	[123]
PCL-based scaffold blended with BCP (70 HA + 30 β-TCP), and added with 1.67% of silver	The 3D scaffold was featured by a highly interconnected and homogeneously distributed porosity, a homogeneous and fine dispersion of BCP, and an increased stiffness	[42]
PCL-based scaffold blended with BCP (70 HA + 30 β-TCP), and added with essential oils	The salt-leaching process formed two types of pores (about 234–208 µm): NaCl determined squared regular pores, whereas NaNO_3_ produced pores with a less defined geometry. The pure PCL specimens slowly lost weight during the immersion	[43]
PCL-based scaffold blended with BCP (70 HA + 30 β-TCP), and added with ~1% of silver	The blending of PCL with BCP provokes a faster weight loss respective to pure PCL	[44]
PCL scaffold with deposition of β-TCP nanoparticle	FESEM analysis demonstrated the random distribution of β-TCP nanoparticles on the surface and the pores of about 300 μm. The XRD revealed the peaks related to PCL and β-TCP	[76]
3D scaffold of PCL and β-TCP, and added or not with MgO nanoparticles	A well-defined microstructure with the pore size of ~500 μm, and the dispersion of the MgO nanoparticles was revealed	[74]
Composite scaffold based on PCL and HA, and incorporated with tetracycline	FESEM images showed the homogeneous distribution of tetracycline on the PCL surface. A high efficiency of its encapsulation was revealed as well as a sustained release over time	[125]
3D scaffold made of PCL and HA, and loaded with ZnO	The higher content of HA makes the constructs more fragile, whereas ZnO was presented in agglomerates	[126]

**Table 4:** Summary of the key literature findings pertaining to antimicrobial properties of PCL-based 3D scaffolds enriched with antibiotics, metal ions, or natural compounds towards different microorganisms.

Scaffold Composition	Microorganisms	Methods	Key Findings	References
PCL loaded with doxycycline	*Escherichia coli* K-12	Agar diffusion test	The drug was successfully loaded in the scaffold and its release produced an inhibition halo of 1 cm	[127]
PCL and HA incorporated with tetracycline	*Escherichia coli* (ATCC^®^ 25922) and *Staphylococcus aureus* (ATCC^®^ 25923)	Agar diffusion test	Despite the drug concentration, the inhibition halo was revealed, but it was more pronounced for *S. aureus* respective to *E. coli*	[125]
Porous membranes of PCL and PLA added with tetracycline	*Staphylococcus aureus* (NCTC 10788) and *Escherichia coli* (NSM59)	Agar diffusion test	A pronounced antibacterial activity of the constructs up to 21 days of incubation, and a larger inhibition halo against *E. coli*	[128]
PCL and β-TCP loaded with microspheres of ceftriaxone	*Escherichia coli*	Agar diffusion test	A sustained drug release was demonstrated with an inhibition halo of 3 cm, after 24 h of incubation	[131]
PCL coated with PLA vancomycin-loaded microspheres	*Staphylococcus aureus* (ATCC^®^ 29213)	Agar diffusion test	A relevant anti-*S. aureus* action was demonstrated over 28 days of incubation	[129]
Coaxial structure based on PCL/PLGA-PVA loaded with erythromycin	*Staphylococcus aureus* (ATCC^®^ 49230)	Agar diffusion test	Higher diameter of inhibition in the growth of *S. aureus* was revealed in relation to erythromycin concentration in the construct	[130]
In situ-added silver nanoparticles on PLGA/PCL	*Staphylococcus aureus* and *Streptococcus mutans*	Agar diffusion test, FESEM images	A wider diameter of inhibition halo for *S. aureus* respective to *S. mutans* was registered, whereas both bacteria attached to the scaffold	[134]
PCL loaded with silver	*Staphylococcus aureus, Escherichia coli, Pseudomonas aeruginosa*, and *Candida albicans*	Agar diffusion test	The antimicrobial effect was different depending on the microorganisms: *C. albicans* was the most susceptible to silver followed by *E. coli*, *S. aureus*, and *Ps. aeruginosa*	[133]
Composites of PCL and BCP enriched with 1.67% of silver	*Staphylococcus aureus* (ATCC^®^ 29213)	Agar diffusion test, adhesion assay	An inhibition halo around the specimen was shown, as well as a reduction in both adhered and planktonic staphylococci	[42]
Composites of PCL and BCP enriched with ~1% of silver	*Staphylococcus aureus* (ATCC^®^ 29213), *Staphylococcus epidermidis* (ATCC^®^ 35984), and *Escherichia coli* (ATCC^®^ 25922)	Agar diffusion test, adhesion assay, FESEM images	An inhibition halo around the silver-enriched sample was shown. A reduction in adherent and planktonic bacteria, and an alteration in their morphology, was revealed. No biofilm formation was shown on the enriched scaffold	[44]
Composites of PCL and BCP enriched with ~1% of silver	*Candida albicans* (ATCC^®^ 10231) and *C. auris* (clinical isolate)	Agar diffusion test, adhesion assay, FESEM images	An inhibition halo around the silver-enriched sample was shown for both strains. A reduction in adherent and planktonic yeasts and a filamentous morphology were revealed. No biofilm formation was shown on the enriched scaffold	[45]
PCL and HA loaded with ZnO	*Staphylococcus aureus* (ATCC^®^ 25923)	Contact with the scaffolds	The release of Zn reduced *S. aureus* load when placed in contact with the scaffold	[126]
Composite 3D membrane of PCL blended with ZnO (from 1% to 7%)	*Staphylococcus aureus* (ATCC^®^ 29923) and *Escherichia coli* (ATCC^®^ 25922)	Agar diffusion test, adhesion assay, FESEM images	Good antibacterial activity on *S. aureus* and *E. coli*, and a reduction in their adhesion to the construct especially at 7% of ZnO	[40]
ZnO nanoparticles added in PCL	*Streptococcus mutans* (KCOM 1504) and *Porphyromonas gingivalis* (KCOM 2804)	Contact with the scaffolds	No significant differences in the bacterial load were obtained by varying the construct composition	[135]
PCL reinforced with copper	*Staphylococcus aureus* and *Escherichia coli*	Agar diffusion test	*S. aureus* (Gram-positive) was more susceptible to copper activity compared to *E. coli* (Gram-negative)	[30]
PCL and gelatin supplemented with chrysin	*Acinetobacter baumannii* (ATCC^®^ BAA-747), *Pseudomonas aeruginosa* (ATCC^®^ 27853), *Staphylococcus aureus* (ATCC^®^ 6538), and *Enterococcus faecalis* (ATCC^®^ 13048)	Agar diffusion test and live/death assay	The scaffold inhibited *A. baumannii*, *Ps. aeruginosa*, *S. aureus*, and *E. faecalis* growth	[142]
CaPs enriched with vanillin	*Escherichia coli* (DH5α)	CFU count after contact with the scaffolds	A reduction in the CFU of *E. coli* only in vanillin presence, as well as an altered morphology of the bacterium	[143]
PCL enriched with 0%, 2%, 4%, and 8% of clove and red thyme	*Candida tropicalis* clinical isolates	Biofilm quantification by crystal violet	The biofilm formation of *C. tropicalis* clinical strains was inhibited when the concentration of the EOs was at 4%	[144]
PCL with cinnamon or thyme at 30%, 40%, and 50%	*Staphylococcus aureus* (ATCC^®^ 29213), *Staphylococcus epidermidis* (ATCC^®^ 35984), and *Escherichia coli* (ATCC^®^ 25922)	Agar diffusion test, adhesion assay, FESEM images	All the concentrations of EOs were able to inhibit the bacteria in growth, adhesion, and biofilm formation. The EO presence modified the bacterial morphology as well	[43]
PCL/PLA enriched with HA and *Nigella sativa* oil at 15, 18, and 20 wt%	*Staphylococcus aureus* and *Escherichia coli*	Agar diffusion test	When *Nigella sativa* was added, the antibacterial activity was obtained only towards *S. aureus* since *E. coli* displayed a natural resistance to this compound	[25]
PCL with graphene oxide at 5% and 7.5%	*Staphylococcus epidermidis* (ATCC^®^ 35984) and *Escherichia coli* (ATCC^®^ 25922)	Live/death assay	The presence of GO increased the number of *S. epidermidis* and *E. coli* dead cells, which was more pronounced at 7.5% of GO	[8]
PCL with chitosan with different molecular weight	*Staphylococcus aureus* (ATCC^®^ 6538) and *S. epidermidis* (ET13)	Adhesion assay and biofilm formation	The addition of chitosan reduced adhesion and biofilm formation of both staphylococci	[10]

**Table 5:** Summary of the key literature findings pertaining to cytocompatibility of mesenchymal stem cells, fibroblasts, and osteosarcoma cells when placed in contact with PCL-based 3D scaffolds.

Scaffold Composition	Cells	Key Findings	References
PCL with CaPs and gelatin	Mesenchymal stem cells	Cells were not impaired in their viability and proliferation, and non-toxic products were released by the scaffold	[121]
PCL enriched with HA and ZnO (1% *w*/*w*)	Mesenchymal stem cells	Cells expressed osteodifferentiation markers and a high calcium deposition was detected in HA presence. Cells colonised the scaffold and differentiated in osteoblasts	[149]
PCL enriched with HA	Mesenchymal stem cells	Cells were anchored and proliferated into the scaffold	[111]
PCL with silica microcapsules	Mesenchymal stem cells	Cells lived in, adhered to, and proliferated into the construct	[147]
PCL functionalised with different concentration of HA	Mesenchymal stem cells	Cells were not hampered in their viability. The greater HA concentration (7%) promoted a superior cell attachment. The cells produced high early-stage differentiation marker on PCL with HA	[120]
PCL/β-TCP or PCL/β-TCP with nano-MgO	Bone marrow mesenchymal stromal cells	Alizarin red S staining revealed the osteoinduction of cells. These cells displayed a long-term viability in MgO presence, as well as an increase in their ALP activity	[74]
PCL coated with PLA vancomycin-loaded microspheres	Rabbit bone marrow-derived mesenchymal stem cells	Eukaryotic cells increased in their amount over time, and some of them, after the attachment, secreted matrix	[129]
Coaxial structure based on PCL/PLGA-PVA loaded with erythromycin	Rat bone marrow stromal cells	Cell growth augmented at erythromycin concentration of 100 μg/mL, but ALP activity decreased at a drug concentration of 500 and 1000 μg/mL	[130]
PCL enriched with different percentages of graphene oxide	Human foreskin fibroblast (HFF-1) cells	Cells adhered to and spread into the construct, for up to 14 days of incubation	[8]
PCL with gelatin and graphene oxide (1% or 2%)	Human gingival mesenchymal stem cells	The scaffold promoted cell adhesion and proliferation	[15]
PCL coated with chitosan, gelatin, and bioactive glass	Fibroblast cells (MG-63)	Human cells were not impaired in viability and proliferation, and the coating enhanced their calcium deposition	[54]
PCL/PLA enriched with gelatin and taurine	Fibroblast cells (MG-63)	The cells were not compromised in their viability and proliferation, after 24 and 72 h	[53]
PCL/PLA and HA, enriched with black curcumin essential oil at increasing concentrations	Human fibroblast cells (CCD-1072-SK)	The oil presence reduced the viability of cells after 24 h of incubation; a lower effect was revealed after 48 h	[25]
Cubic-shaped PCL and chitosan	Mouse murine fibroblast cells (L929)	Cells displayed a higher viability and maintained their phenotype	[10]
Composites of PCL and HA incorporated with tetracycline	Fibroblast cells (L929)	Cells were viable and the tetracycline concentration did not affect their viability	[125]
PCL	Mouse murine fibroblast cells (L929)	Cells were not impaired in their viability and morphology, and they preserved the spherical shape	[122]
CaPs enriched with vanillin	Fibroblast-like cells (ATCC^®^ L929) and human osteoblast-like cells (ATCC^®^ MG-63)	Fibroblasts were not impaired in viability when vanillin was present and were uniformly distributed. Also, the viability of osteoblasts was promoted within a short time of incubation	[143]
PCL with β-TCP nanoparticle deposition	Pre-osteoblasts (MC3T3-E1)	Cells showed high viability, proliferation, and adhesion as well as an increase in ALP activity and mineralisation	[76]
Composites based on PCL and Zn (at 1, 2, or 3 wt%)	Pre-osteoblasts (MC3T3-E1)	A greater number of live cells was recorded at 2–3 wt% of Zn respective to pure PCL or the one with 1 wt% of Zn	[86]
PCL with the copolymer Inulin-g-poly(D,L)lactide	Human fibroblasts (ATCC^®^ MRC-5-CCL-171) and human adipose-derived mesenchymal stem cells	An adequate cytocompatibility towards fibroblasts was proved. Additionally, a 100% viability of human adipose-derived mesenchymal stem cells and their attachment to the biomaterial surface was revealed, as well as their high production of differentiation markers	[124]
PCL loaded with silver	Human dermal fibroblasts	High cytocompatibility of PCL added with silver but only at low concentrations (from 2.5% to 1%)	[133]
Pure PCL or PCL blended with BCP at 20% or 30%	Osteoblast cell lines (MC3T3-E1, subclone 4)	Cells attached and proliferated with multi-layers but also differentiated as a result of an increase in ALP activity and in OCN gene expression	[123]
Composites of PLA and PCL	Osteoblast cell lines (MC3T3-E1, subclone 4)	Cells proliferated in the scaffold, while displaying a good cytocompatibility, at 2 and 3 days of incubation	[47]
ZnO nanoparticles added in PCL constructs	Osteoblast cell lines (MC3T3-E1, subclone 4)	No significant difference in the viability of cells was observed for pure PCL compared to the modified one	[135]
In situ-added silver nanoparticles on PLGA/PCL scaffolds	Osteoblast cell lines (MC3T3-E1, subclone 14)	Cells cultured with the scaffolds reported higher proliferative capability when silver was at the lowest concentration. FESEM images showed cell attachment to the scaffolds as well as the presence of extended filopodia. Furthermore, an increase in ALP and mineralisation was demonstrated	[134]
PCL reinforced with copper	Human osteoblastic-like cells (MG63) and mouse mesenchymal stem cells	Mesenchymal stem cells were not impaired in either viability or migratory capability. Also, they showed increased expression of osteoblast differentiation markers, as well as calcium deposition	[30]
PCL and HA loaded with ZnO	Human foetal osteoblast cell line (HFOb 1.19)	Zn presence enhanced ALP activity and promoted cells’ calcium deposition. However, it reduced their viability	[126]
PCL–chitosan enriched with Zn at increasing concentrations	Mouse fibroblast cell (NIH-3T3 lines)	Only the lower Zn concentrations (10 and 20) allowed a 100% viability of cells with an increase in cellular attachment and proliferation	[150]
PCL blended with BCP and enriched with silver at 1.67% or essential oils	Human osteosarcoma cell—Saos-2	Specimens were not toxic for osteoblasts and the addition of BCP did not impair their viability. Cells adhered, proliferated, and did not alter their morphology. Silver at 1.67% impaired cell viability, as well as essential oils at 40–50%	[42,43]
PCL and β-TCP loaded with ceftriaxone microspheres	Human osteosarcoma cell—Saos-2	An increased number of viable cells was determined within 28 days of incubation, with the attachment and spreading of osteoblasts	[131]
PCL blended with BCP and enriched with silver at ~1%	Human osteosarcoma cell—Saos-2	Pure PCL was not toxic for Saos-2 cells, whereas only the lowest silver concentration allowed cellular survival and proliferation	[44]
PCL and blended with BCP, and enriched with silver at ~1%	Human osteosarcoma cell—Saos-2	The scaffolds were able to promote calcium deposition by SaoS-2 cells	[45]
PCL and HA at different percentages	Human osteoblast cell line (MG-63)	The biomaterial was non-toxic and permitted the colonisation by the cells, which maintained their spherical shape. Their calcium deposition was also demonstrated	[80]
PCL and HA at increasing percentages	Human osteoblasts	Cells proliferated into, attached to, and covered the surface, while displaying a flat poliedric morphology	[115]
PCL blended with HA	Human osteoblast cell line (MG-63)	Cells successfully proliferated, deposed calcium, and upregulated the expression of genes involved in differentiation, mainly in HA presence	[117]
PCL blended with 1% of HA	Human osteoblasts (HOB-Promocell C-12720)	HA addition increased the viability, proliferation, and calcium deposition of human osteoblasts, which were featured by filopodia	[118]
PCL, HA, and chitosan	Human osteosarcoma cells (MG-63)	Osteoblasts were viable and their proliferation increased proportionally to HA concentration. Cells expressed different genes involved in osteogenesis	[16]
PCL, gelatin, and nanohydroxyapatite	Human osteoblasts	Cells increased their viability and proliferation and displayed a polygonal-shaped morphology	[4]
Biphasic PCL/HA nanofibrous scaffold	Immortalised myoblast cell line (C2C12)	Cells attached, spread, and increased ALP activity mainly when HA was added	[119]

## Data Availability

Not applicable.

## Abbreviations

3D, Three-dimensional; AgVO, Silver vanadate oxide; ALP, Alkaline phosphatase; ATCC^®^, American Type Culture Collection; BBG, Borate bioactive glass; BCP, Biphasic calcium phosphate; BMPs, Bone morphogenetic proteins; CaPs, Calcium phosphates; Cu, Copper; DCM, Dichloromethane; DLP, Digital light processing; DMF, Dimethylformamide; EBP, Extrusion-based printing; EO, Essential oil; ES, Electrospinning process; FDM, Fused deposition modelling; FESEM, Field emission scanning electron microscopy; GO, Graphene oxide; HA, Hydroxyapatite; HLh, Halomonas levan; MES, Melt electrospinning; MEW, Melt electrospinning writing; MgO, Magnesium oxide; MTT, 3-(4,5-dimethylthiazol-2-yl)-2,5-diphenyltetrazolium bromide) tetrazolium; NaCl, Sodium chloride; NaNO_3_, Sodium nitrate; OCN, Osteocalcin; PCL, Polycaprolactone; PHB, Poly (3-hydroxybutyrate); PLA, Poly (lactic acid); PLGA, Poly (lactic-co-glycolic) acid; PVA, Polyvinyl alcohol; Saos-2, Human osteosarcoma; SC/PL, Solvent casting/porogen leaching; SEM, Scanning electron microscopy; SLA, Stereolithography; SLS, Selective laser sintering; TIPS, Thermally induced phase separation; UV, Ultraviolet; XRD, X-ray diffraction; Zn, Zinc; ZnO, Zinc oxide; β-TCP, β-tricalcium phosphate.
